# An A-E assessment of post-ICU COVID-19 recovery

**DOI:** 10.1186/s40560-021-00544-w

**Published:** 2021-03-20

**Authors:** Matthew Cadd, Maya Nunn

**Affiliations:** Intensive Care Unit, Royal Sussex County Hospital, Brighton, BN2 5BE UK

**Keywords:** COVID-19, Recovery, Critical care, Mental health, Dyspnoea, Anosmia, Nutrition, Embolism

## Abstract

The COVID-19 global pandemic has placed unprecedented strain on healthcare and critical care services around the world. Whilst most resources have focused on the acute phase of the disease, there is likely to be an untold burden of patients chronically affected.

A wide range of sequelae contribute to post intensive care syndrome (PICS); from our current knowledge of COVID-19, a few of these have the potential to be more prevalent following critical care admission. Follow-up assessment, diagnosis and treatment in an increasingly virtual setting will provide challenges but also opportunities to develop these services. Here, we propose an A to E approach to consider the potential long-term effects of COVID-19 following critical care admission.

**A**nxiety and other mental health diagnoses

**B**reathlessness

**C**entral nervous system impairment

**D**ietary insufficiency and malnutrition

**E**mbolic events

Developing strategies to mitigate these during admission and providing follow-up, assessment and treatment of persistent multiple organ dysfunction will be essential to improve morbidity, mortality and patient quality of life.

## Background

COVID-19 has caused a global surge in critical care demand not previously seen in history. COVID-19 has the potential to have a protracted course, a significant number of patients show symptoms lasting more than 8 weeks [1, 2]. As more COVID-19 critical care survivors begin their recovery, there may be some unique features of post COVID-19 intensive care syndrome that will require ongoing assessment and follow-up. Here, we aim to provide some wider considerations of these based on the limited data from current post COVID-19 research and also extrapolated from longer term data from other critical care populations and notably previous respiratory virus pandemics.

## Main text

**A**nxiety, depression and post-traumatic stress disorder (PTSD) are frequently seen following critical care admission; in pre-pandemic studies, 35–45% of patients met diagnostic criteria for anxiety 12 months following discharge and 18% meeting diagnostic criteria for all three diagnoses [3, 4]. Risk factors for developing mental health disorders following critical care admission include longer periods of mechanical ventilation and delirium [5]. Given COVID-19 patients are requiring longer periods of mechanical ventilation and sedation, combined with dexamethasone being used for treatment, these patients are predisposed to higher rates of delirium and subsequent mental health disorders [6, 7]. The effect of social isolation on patients, before, during and after critical care admission should be taken into account; a small number of studies have shown that loneliness and resulting depression has increased during the pandemic [8, 9].

Additionally, there could be a direct neurological effect of the SARS-CoV-2 as was noted with SARS-CoV and MERS or indirect as part of the systemic sequalae of the disease [10]. Either way, there are reports of widespread neurological effects of COVID-19; a study from Wuhan found patients with more severe COVID-19 had higher rates of neurological outcomes (45% compared to 36% in those with less severe disease) [11]. A biological cause for mental health problems following infection should not be overlooked.

Critical care diaries have been shown to reduce PTSD; these would not only provide a story of their admission as documented by staff but could be used to communicate messages from relatives unable to visit during admission [12].

Offering critical care follow-up to discuss specific concerns and screening for mental health disorders, in either a clinic or primary care setting, using a suitable questionnaire such as the GHQ-28, will be particularly important in post critical care COVID-19 patients [13–15].

**B**reathlessness, reduced exercise tolerance and impaired pulmonary function were common amongst patients following the previous SARS epidemic and have been shown to cause considerable morbidity following COVID-19 [16, 17]. Anaemia, respiratory muscle dysfunction, deconditioning, pulmonary embolism and anxiety can all contribute to the often-multifactorial nature of post critical care breathlessness [18]. Early planning for rehabilitation whilst patients are still in critical care is important to have the greatest impact on recovery [19].

Pulmonary rehabilitation (PR) aims to increase functional capacity, reduce symptoms and improve quality of life [18]. PR is multifaceted and requires patient education, psychosocial support, exercise prescription and medical optimisation.

Virtual PR has shown some early promise in COVID-19 patients, including video classes, booklets and telephone appointments [20]. Remote assessment with a 6-min walk test using a mobile application combined with a home pulse oximetry could be used to help stratify patients who need further input and face-to-face assessment [21, 22].

**C**entral nervous system impairment and particularly chemosensory disturbance has shown to be a common symptom of COVID-19. A recent meta-analysis has shown olfactory dysfunction and taste dysfunction to be present in 38.5% and 30.4% of COVID-19 patients, respectively [23]. As opposed to other viral causes of anosmia or hyposmia, it is thought that COVID-19 is neurotropic rather than causing nasal congestion [24, 25].

Although some studies report anosmia as being indicative of less severe disease and lower mortality, a significant number of critical care patients still report chemosensory disturbance [26]. Critical care acquired weakness and loss of muscle mass may be exacerbated by lack of enjoyment in eating and subsequent malnutrition due to absent taste and smell [27].

Although there is limited evidence in COVID-19 to date, olfactory training is a treatment that can be completed remotely and has shown to be effective for sensorineural olfactory loss [28–30].

**D**ietary intake is an important consideration for all critical care patients. COVID-19 has presented its own challenges for providing nutrition to patients in critical care including debate around if nasogastric tube insertion is aerosol generating and potential challenges of enteral feeding in proned patients [31, 32].

To date there have been no studies looking specifically at the nutritional status of COVID-19 critical care patients. As previously discussed, COVID-19 patients have required a prolonged length of stay compared to other critical care patients. This combined with the hypercatabolic state it induces put this group at higher risk of malnutrition and subsequent complications [6, 33]. Inadequate nutritional intake in critical care patients has shown to increase mortality and time to physical recovery [34].

Post extubation dysphagia, critical care acquired weakness, anosmia and ageusia are factors that will make it challenging for patients to meet their nutritional requirements. A nutritional plan, with regular assessment and progressive physical exercise should be the mainstay of recovery [35].

**E**mbolic and thrombotic events have been more prevalent in the COVID-19 critical care population, 31% and 5% of patients have been shown to have venous and arterial thromboembolisms, respectively [36–39]. COVID-19 has been shown to cause profound hypercoagulability, complement activation and cytokine storm [40, 41], which can lead to widespread embolic events in the arterial and venous systems. Longer periods of immobility, staffing constraints and isolation restrictions may further increase clot risk [42].

Pulmonary embolism, cerebrovascular accident, myocardial infarction and limb ischaemia can cause significant morbidity and mortality both immediately and chronically. Early adequate therapeutic anticoagulation and recognition of the aforementioned diagnosis will help to reduce clot burden whilst not increasing bleeding incidence [43, 44].

Although no long-term data currently exists for embolic risk post COVID-19 (both in patients with confirmed VTE and those without), there should be risk stratification for further thrombosis and haemorrhagic risk and consideration of longer treatment or empirical anticoagulation [45].

## Conclusion

COVID-19 has so far shown to be a multi-organ disease affecting all ages. Although PICS is well-recognised in critical care survivors, there is limited evidence of the chronic effects of COVID-19 in post critical care patients at present [46]. We believe PICS will be prevalent following COVID-19 but the symptoms and diagnoses discussed here may be unique to COVID-19 and should be considered following critical care discharge.

As public health restrictions continue to be dynamic, the medium for providing assessment should minimise face to face contact so as to maintain some consistency in service provision. A wide-ranging multidisciplinary team both in primary and secondary care will be required to address the aforementioned sequalae and additional ones not discussed here. Current guidelines suggest rehabilitation assessment and initiation of treatment should start whilst patients remain in critical care [47].

Treatment of these conditions are wide ranging from psychological to pharmacological. As more data emerges about specific complications in this patient group, treatment strategies and guidelines will be implemented and updated. More extensive guidelines have been produced elsewhere highlighting current best practice for on-going assessment and treatment of post-acute COVID-19 in the community setting [20].

Multi-centre longitudinal studies are currently underway to assess the long-term impact of COVID-19 and the needs of COVID-19 patients who have been hospitalised. Analysis of these large studies should guide best practice [48].

The unifying aim of assessing each of the aspects of post COVID-19 care is to adequately treat patients to reduce morbidity and mortality, improve quality of life and help enable patients to return to their previous baseline of physical and mental functionality.

## Data Availability

We have not collected or analysed any data.

## Abbreviations

COVID-19: Coronavirus disease 2019
PICS: Post intensive care syndrome
PPE: Personal protective equipment
PTSD: Post-traumatic stress disorder
GHQ-28: General Health Questionnaire 28
SARS: Severe acute respiratory syndrome
PR: Pulmonary rehabilitation
VTE: Venous thromboembolism
